# STRENGTHENING AGE-FRIENDLY COMMUNITIES THROUGH CAPACITY BUILDING TO ADDRESS BEHAVIORAL HEALTH CONCERNS

**DOI:** 10.1093/geroni/igz038.1139

**Published:** 2019-11-08

**Authors:** Bronwyn Keefe, Rita Kostiuk

**Affiliations:** Boston University, School of Social Work, Boston, Massachusetts, United States

## Abstract

The Center for Aging and Disability Education and Research at Boston University in collaboration with the Age-Friendly New Bedford received funding from Tufts Health Plan Foundation to reduce the stigma of mental illness and increase awareness of the effects of social isolation in the community. In order for older adults to be fully engaged in community life, behavioral health concerns need to be addressed with a focus on social isolation, depression, and substance use. Many Age Friendly efforts don’t address these issues even though significant numbers of older adults are impacted. Without a community-wide capacity building effort, behavioral health issues among older adults often fade into the background. We developed and implemented a 3-tiered approach to incorporating behavioral health into an Age Friendly initiative. In the first tier, we focused on increasing awareness of the impact of behavioral health concerns and stigma by creating an anti-stigma campaign in multiple languages. The second tier focused on holding workshops for older adults on behavioral health related issues. The third tier provided training to key stakeholders, including aging service providers, clergy, first responders, and resident coordinators focusing on the need to effectively identify and respond to older adults with behavioral health concerns. Using the Depression Stigma Scale, we measured perceived stigma among older adults pre and post workshop participation. We found statistically significant changes in how older adults perceive depression after participation in the workshop. Training results were also statistically significant with gains pre-post training in key competency areas.

